# Special Issue: Avian Adenoviruses

**DOI:** 10.3390/v14040680

**Published:** 2022-03-25

**Authors:** Anna Schachner, Michael Hess

**Affiliations:** 1 Christian Doppler Laboratory for Innovative Poultry Vaccines (IPOV), University of Veterinary Medicine, 1210 Vienna, Austria; anna.schachner@meduniwien.ac.at; 2 Clinic for Poultry and Fish Medicine, Department for Farm Animals and Veterinary Public Health, University of Veterinary Medicine (Vetmeduni Vienna), 1210 Vienna, Austria

For years, research on avian adenoviruses, here fowl adenoviruses (FAdVs), received less attention, mainly due to limited clinical relevance in poultry production. However, along with a change in this situation in the last two decades, a paradigm shift has occurred, which has paved the way towards intensified research and scientific activities [1]. Today, FAdVs are important pathogens in the field, causing diseases with substantial impact on poultry health and welfare. Various reviews published in recent years provide a record of this development [2,3,4,5]. The number of submitted manuscripts devoted to FAdVs and the avid resonance to this Special Issue also underline the interest and topicality of the subject. In that regard, the guest editors wish to thank everyone who contributed, either by preparing an article or assisting in the review process, to the successful realization of this volume.

For a long time after the first discovery of FAdVs in the middle of the last century, single outbreaks of associated diseases were reported without major spread, except for hepatitis-hydropericardium syndrome (HHS), which appeared in 1989. Since then, HHS and the causative agent FAdV-4 became endemic in certain countries. The disease gained even more importance with its recent emergence in China, where it also spread to other poultry species, especially ducks, kept for production purposes [6,7]. Together with an increasing record of outbreaks of inclusion body hepatitis (IBH) occurring worldwide, the demand to protect birds by vaccination became evident. With a need to cover both HHS and IBH, this also increased the number of serotypes attributed to pathologies, complicating vaccine development on the side of much-desired cross-protection. Field data on this challenging question are very limited, and this Special Issue contributes with a detailed review on the Korean situation monitored over a period of 15 years [8]. In this study, not only a switch in serotypes in the field was noticed in response to pre-existing immunity, but the findings also pointed towards a lack of cross-protection between different serotypes. With the increasing importance of HHS and IBH, it can be predicted that more of such studies will be published in the future. In parallel, new vaccine technologies will be applied in order to increase the panel of available vaccines, for example, live vaccines developed by creating recombinant viruses [9,10]. This will also contribute to the understanding of the underlying mechanisms of pathogenicity, as strain variation within FAdV serotypes is quite substantial. In this context, perception of the role of hexon is very new and it remains to be elucidated whether this can be transferred to other serotypes as well and if it can even be narrowed down to a single amino acid change [11,12]. Unsurprisingly, other genes are participating in pathogenicity as well, especially fibers as reported for FAdV-4 [13,14,15]. In a contribution to this Special Issue, Grafl et al., 2022, for instance, show that FAdV-1 pathogenicity is driven by factors outside hexon and that even strains with near-identical genomes possess very different in vivo properties [16]. A somewhat slower replication cycle in vitro is correlated with the pathogenicity in vivo. Remarkably, in another study of this Special Issue, it was shown that the replication cycle in vitro not only differs between FAdVs but also influences the expression of MHC-I [17]. Using FAdV-9, it was further shown that this feature, which is well-known from human adenoviruses, is mediated by the left end of the genome. This also underlines the complexity of the FAdV genomes, which needs further research to fully elucidate the host–pathogen interaction on the level of individual genes. While whole genome sequences are mandatory for such detailed analyses, they may be insufficient to assign serotypes, for which cross-neutralization tests remain the unrivaled gold standard, as shown for FAdV-5, the sole serotype representative of fowl aviadenovirus species B [18]. In addition to important implications for taxonomy, such studies also contribute to our understanding of FAdV evolution.

Aligning neatly with the spectrum of published studies, and following up on some of the most compelling questions in the research field, the contents of the Special Issue showcase the diverse range of subjects investigated in FAdVs. It is with great pleasure that we bring to you the final product of those collective efforts and we hope you enjoy an interesting read.
